# Modulators of the Balance between M1 and M2 Macrophages during Pregnancy

**DOI:** 10.3389/fimmu.2017.00120

**Published:** 2017-02-09

**Authors:** Yong-Hong Zhang, Ming He, Yan Wang, Ai-Hua Liao

**Affiliations:** ^1^Family Planning Research Institute, Center for Reproductive Medicine, Tongji Medical College, Huazhong University of Science and Technology, Wuhan, P.R. China; ^2^Department of Obstetrics and Gynecology, Maternal and Child Health Hospital of Hubei Province, Wuhan, P.R. China

**Keywords:** macrophage, polarization, pregnancy, maternal-fetal interface, GM-CSF, HLA-G, notch signal, Tim-3

## Abstract

Macrophages are a subset of mononuclear phagocytes of the innate immune system with high plasticity and heterogeneity. At the maternal–fetal interface, macrophages are present in all stages of pregnancy and involved in a variety of activities, including regulation of immune cell activities, decidualization, placental cell invasion, angiogenesis, parturition, and postpartum uterine involution. The activation state and function of uterine–placental macrophages are largely dependent on the local tissue microenvironment. However, disruption of the uterine microenvironment can have profound effects on macrophage activity and subsequently impact pregnancy outcome. Thus, appropriately and timely regulated macrophage polarization has been considered a key determinant of successful pregnancy. Targeting macrophage polarization might be an efficient strategy for maintaining maternal–fetal immune homeostasis and a normal pregnancy. Here, we will review the latest findings regarding the modulators regulating macrophage polarization in healthy pregnancies and pregnancy complications, which might provide a basis for macrophage-centered therapeutic strategies.

## Introduction

During pregnancy, the maternal immune system is greatly challenged by the semiallogeneic fetus. Instead of immune-mediated rejection, maternal immune adaptation occurs systematically and locally, especially at the maternal–fetal interface. The maternal–fetal interface is a unique microenvironment including three distinct components: the fetal-derived trophoblast, maternal-derived decidual stromal cells, and immune cells. Although the immune cell composition undergoes dramatic changes as gestation progresses, these changes are necessary for maternal–fetal tolerance and healthy pregnancy. Macrophages, approximately 20–25% of the total decidual leukocytes and the predominant subset of human antigen-presenting cells at the maternal–fetal interface, are in close proximity to the extravillous trophoblast and in the vicinity of spiral arteries. Therefore, they are proposed to be involved in several processes required for a successful pregnancy, including immune tolerance, trophoblast invasion, tissue and vascular remodeling, embryo growth, and initiation of parturition (1). All of these functions are manifestations of macrophage plasticity and heterogeneity, namely, the M1 and M2 subtypes (2). The M1 subtype refers to the classically activated macrophage and displays the capacity to present antigens to the adaptive immune system. With high expression of major histocompatibility complex class II, CD80, CD86, and IL-12, M1-polarized macrophages are more effective at antigen clearance and switching T-cell responses toward T helper-1 immune response (3). Compared to M1 phenotype, M2 populations are alternatively activated. Characterized by typical M2-associated markers (e.g., CD163, CD206, CD209, and IL-10), M2 cells have immunosuppressive capacities, contribute to tissue remodeling, and promote Th2 or antibody-mediated immune responses (4) (Table 1).

**Table 1:** **Phenotype and function of macrophage subsets**.

	Subtypes	
	M1	M2
Inducers	IFN-γ, LPS, GM-CSF, oxidative, fatty acid, HMGB1	IL-4, IL-10, IL-13, TGF-β, M-CSF, AMP, GC
Transcription factors	NF-κB, STAT1, IRF1, IRF5, HIF-1α, KLF6	STAT3, STAT6, IRF4, KLF4, PPARγ, cMaf, cMyc
Cytokines	NO, TNF-α, IL-1β, IL-6, IL-12, IL-23	IL-10, TGF-β
Chemokines	CXCL9, CXCL10, CXCL11	CCL17, CCL18, CCL22
Metabolic enzymes	iNOS, gp91phox and p22phox, ferritin, CP, DMT-1, Narmp-1	Arg-1, Arg-2, ODC, SMO, HO-1, Fpn, TfR
Cell marker	CD80, CD86, TLR2, TLR4, MHC II	CD206, CD163, CD209, CD301, Fizzl, Ym1/2
Functions	Pro-inflammatory, microbicidal activity, clearance of pathogen	Anti-inflammatory, immune regulators, tissue repair

*AMP, adenosine monophosphate; Arg, arginase; CCL, chemokine (C-C motif) ligand; CP, ceruloplasmin; CXCL, chemokine (C-X-C) ligand; DMT, divalent metal transporter; Fizz1, resistin-like α; Fpn, ferroportin; GC, glucocorticoids; GM-CSF, granulocyte macrophage colony-stimulating factor; HIF, hypoxia inducible factor; HMGB1, high-mobility group box 1; HO-1, hemeoxygenase-1; iNOS, inducible nitric oxide synthase; IFN-γ, interferon-gamma; IL, interleukin; IRF, interferon regulatory factor; KLF, Kruppel-like factor; LPS, lipopolysaccharides; MHC, major histocompatibility complex; M-CSF, macrophage colony-stimulating factor; NF-κB, nuclear factor κB; NO, nitric oxide; Nramp, natural resistance-associated macrophage protein; ODC, ornithine decarboxylase; PPAR, peroxisome proliferator-activated receptors; SMO, spermidine oxidase; STAT, signal transducer and activator of transcription; TNF-α, tumor necrosis factor alpha; TGF-β, transforming growth factor beta; TfR, transferrin receptor; Ym1, chitinase 3-like 3*.

Tissue macrophages are deposited during embryonic development of originating from yolk sac cells as early as embryonic day 8.5 and from fetal liver after gastrulation (5). In homeostatic conditions, macrophages are maintained by self-renewal (6). Under inflammatory condition, the embryonically derived macrophages could be partially replaced by bone marrow-derived monocytes (7). Macrophages are abundant in the uterus, being the second most abundant endometrial leukocyte population and the predominant myometrial leukocyte population. The numbers of macrophages fluctuate during the estrus cycle and menstrual cycle, which are driven by estrogen and progesterone (8–10). Immediately after copulation, more macrophages are attracted to the endometrium by seminal fluid (11), indicating that a large number of macrophages are necessary to sustain the pregnancy. More evidence for the importance of macrophages was recently provided by Care et al. (12), who reported that specific depletion of macrophages resulted in implantation failure. Furthermore, decidual macrophages are a heterogeneous population with diverse phenotypes that facilitate adaptive responses to the ever-changing environment. Although it has been shown that decidual macrophages do not belong to either of the M1 and M2 subsets (13), some studies have suggested that M2 macrophages or M2 subgroups are the predominant phenotype in the decidua (14).

**Figure 1:**
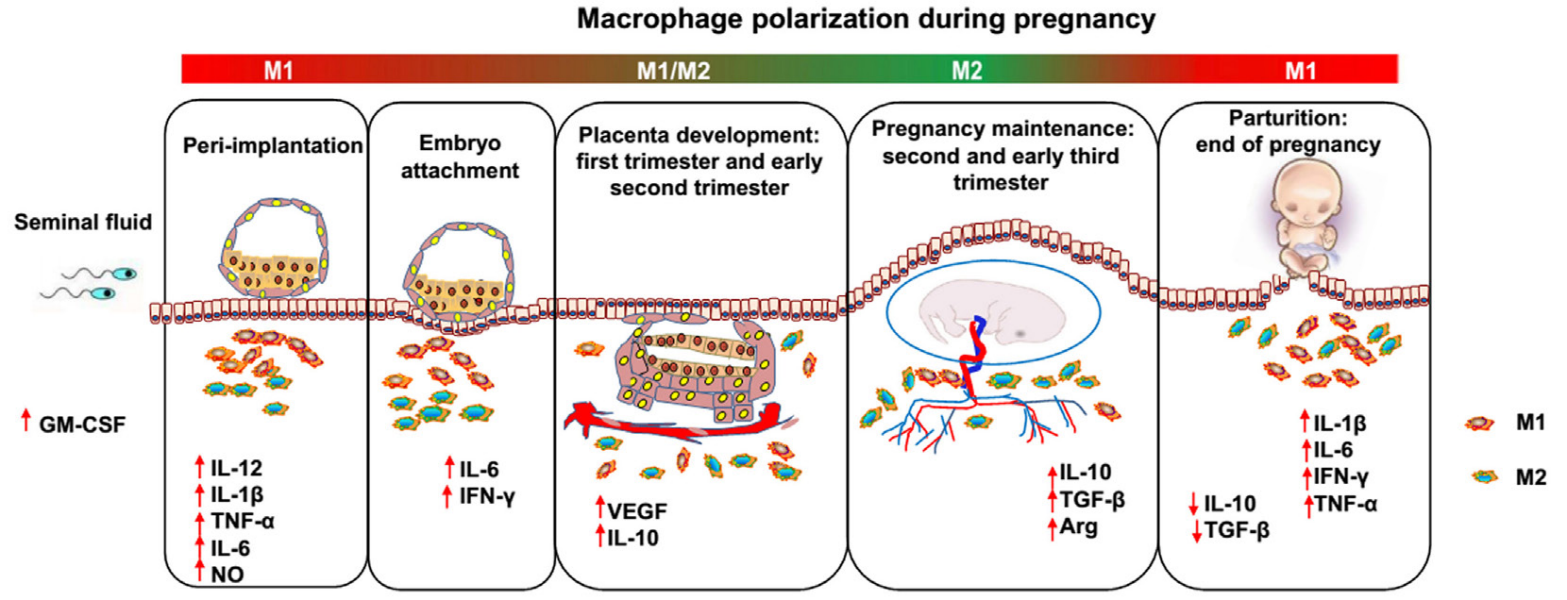
**Dynamics between M1 and M2 macrophages along pregnancy**. During the different phases of gestation, macrophages undergo dynamic changes, predominantly displaying the M1 or M2 phenotype. After coitus, granulocyte macrophage colony-stimulating factor levels are increased by transforming growth factor beta (TGF-β) in the seminal fluid and promote M1 activation. In the peri-implantation period, activated M1 macrophages produce inflammatory cytokines and mediators, such as interleukin (IL)-6, IL-1β, tumor necrosis factor alpha, and nitric oxide, inducing pro-inflammatory responses and promoting embryo attachment to the decidua. As the trophoblast invades the uterine stroma, decidual macrophages initiate an M1/M2 profile until the early phase of the second trimester of pregnancy, displaying both the pro- and anti-inflammatory phenotype, which endows the host with the ability to promote trophoblast invasion and vascular remodeling and prevent rejection of the embryo. Subsequently, in order to allow fetal development, more progesterone is produced, and an M2-dominant environment is established in the uterus until the end of pregnancy, which includes downregulation of inflammatory mediators, increased generation of anti-inflammatory cytokines (e.g., IL-10 and TGF-β), and phagocytosis of apoptotic debris. Finally, M1 macrophages predominate over the M2 subset again during the period of parturition, which is considered an inflammatory event. Accumulated M1 macrophages promote the contraction of the uterus, expulsion of the baby, ejection of the placenta and uterine involution.

Pregnancy has been proposed as a dynamic and highly regulated immunologic process (15). Therefore, successful pregnancy requires that the macrophage activation status remains appropriately regulated throughout pregnancy (Figure 1). During the window of the implantation period, macrophages are induced toward M1 activation (16). However, as trophoblasts attach to the endometrial lining and invade the uterine stroma, macrophages switch to a mixed M1/M2 profile (16). The mixed polarization pattern runs through the first trimester and the early phase of the second trimester of pregnancy when the uterine vasculature undergoes remodeling in order to establish an adequate placental–fetal blood supply. After placentation is complete, the macrophages shift toward M2 polarization, which prevents rejection of the fetus and allows fetal growth until parturition. Parturition, which is considered a pro-inflammatory event, is preceded by an accumulation of M1 macrophages in the uterus (17). This inflammatory process promotes the contraction of the uterus, expulsion of the baby, ejection of the placenta, and uterine involution. However, inappropriate macrophage polarization, regardless of when it occurs, is usually associated with abnormal pregnancies, such as spontaneous abortion (18), preterm labor (PTL) (19), preeclampsia (PE) (20), fetal intrauterine growth restriction (IUGR) (21), and intrauterine parasitic infections (22). Therefore, further insight into macrophages would be of great benefit to reproductive immunology (23). However, despite the important roles of macrophages during pregnancy, little is known about the factors responsible for triggering macrophage differentiation and polarization (24). In the current review, we discuss studies that have modulated macrophage polarization in order to provide an overview of potential targets that may promote macrophage homeostasis and normal pregnancy.

## Macrophage Polarization

Macrophage polarization is triggered by signals present in the surrounding environment, accompanied by a set of signaling pathways, transcriptional and posttranscriptional regulatory networks (25). At the most fundamental level, M1/M2 polarity arises from arginine metabolism *via* two enzymatic pathways [inducible nitric oxide synthase (iNOS) and arginase] that are distinct and antagonistic (2, 26). The M1 subtype is a product of the iNOS pathway, whereas M2 is the product of the arginase pathway (2). The factors that determine which pathway is dominant are based on the surrounding signals that the macrophages are exposed to and the available arginine pool (2, 27). Therefore, the final activation status of macrophage polarization is ultimately decided by the surrounding milieu. Various surrounding signals participate in macrophage polarization, including adaptive immunity and microorganism-derived molecular patterns, such as lipopolysaccharides (LPS), cytokines, and growth factors released by the injured tissue. Generally, polarized M1 and M2 macrophages are induced and represent the two extremes of a broad spectrum of differentiation states. However, this does not alter their terminal differentiation status. Once M2 macrophages are exposed to M1 signals, or *vice versa*, “re-polarization” of already differentiated macrophages can occur, which might be more evidence of their highly functional plasticity. Furthermore, this re-education of macrophages is currently under investigation for therapeutic purposes (28). Therefore, a mixed phenotype representing a superposition of the M1 and M2 phenotypes might exist; this was confirmed by findings which suggested that macrophages adopt a mixed phenotype dependent on the relative strength of the stimuli and that cells progress toward an M2 phenotype over time (29). Therefore, macrophage reprogramming by combined activation signals might be dependent on the initial polarization state and doses of stimulation.

Interferon-gamma (IFN-γ), either alone or in combination with other stimulants, including LPS, tumor necrosis factor alpha (TNF-α), and granulocyte macrophage colony-stimulating factor (GM-CSF), induces M1 macrophage polarization (4, 30). In addition, IFN-γ and LPS are widely used to induce M1 polarization *in vitro*. IFN-γ induces downstream phosphorylation of signal transducers and activators of transcription-1 (STAT1) by Janus kinases (JAK). LPS specifically activates toll-like receptor (TLR)-4, which can affect the mitogen-activated protein kinase pathway, the interferon regulatory factor pathway, and the nuclear factor κB (NF-κB) pathway by inactivating the inhibitor of NF-κB kinase (IKK)-2. Furthermore, the NF-κB pathway has also been implicated in the regulation of STAT1 activity in M1 macrophages. When NF-κB activity is diminished through deletion of IKK-2, STAT1 activity is enhanced in mouse macrophages (31). The enhanced STAT1 activity subsequently contributes to M1 polarization, with the production of nitric oxide (NO) and the secretion of pro-inflammatory cytokines, such as interleukin (IL)-1β, IL-6, IL-12, IL-23, and TNF-α (4, 32). The Notch signaling pathway is also involved in LPS-TLR-4-induced expression of inflammatory M1 macrophage cytokines. LPS treatment activates the Notch pathway by a c-Jun N-terminal kinase (JNK)-dependent pathway (33), which enhances NF-κB phosphorylation (34) and pro-inflammatory cytokine secretion (IFN-γ and TNF-α) (35).

In contrast, M2 macrophage polarization can be achieved *in vitro* by macrophage colony-stimulating factor (M-CSF), IL-4, IL-10, IL-13, IL-33, and/or transforming growth factor beta (TGF-β). Both IL-4 and IL-13 activate the JAK–STAT pathway, leading to the activation of STAT6, which is essential for the expression of M2 macrophage markers (36). In M2 macrophages, the production of NO and pro-inflammatory cytokines is diminished, but anti-inflammatory cytokines, such as TGF-β and IL-10, are produced. Although the effects of ILs on macrophages are fairly well studied, TGF-β remains to be the most puzzling cytokine in regard to its effects on macrophages. Nevertheless, TGF-β plays an important role in the pathogenesis of many diseases where macrophages play a key role as well. The relationship between macrophages and TGF-β has attracted the attention of researchers since the description of this growth factor (37). Initially, TGF-β was found to be a potent immunosuppressive and “macrophage-deactivating” agent (38). Later, the role of TGF-β in the function of macrophages was described. The best studied is the role of TGF-β in the recruitment and development of tumor-associated macrophages (TAMs) (M2 phenotype) (39, 40). It induces an M2-like phenotype by activating the canonical Smad2/3-mediated signaling as well as Smad1/5-mediated signaling (37). Activities of TAM regulated by TGF-β stimulate proliferation of tumor cells and lead to tumor immune escape. Therefore, it is clear that understanding of molecular mechanisms of TGF-β–TAM interaction is highly important for therapeutic targeting of TGF-β or macrophages (37).

## What Modulates the Balance Between M1 and M2 Macrophages During Pregnancy

**Figure 2:**
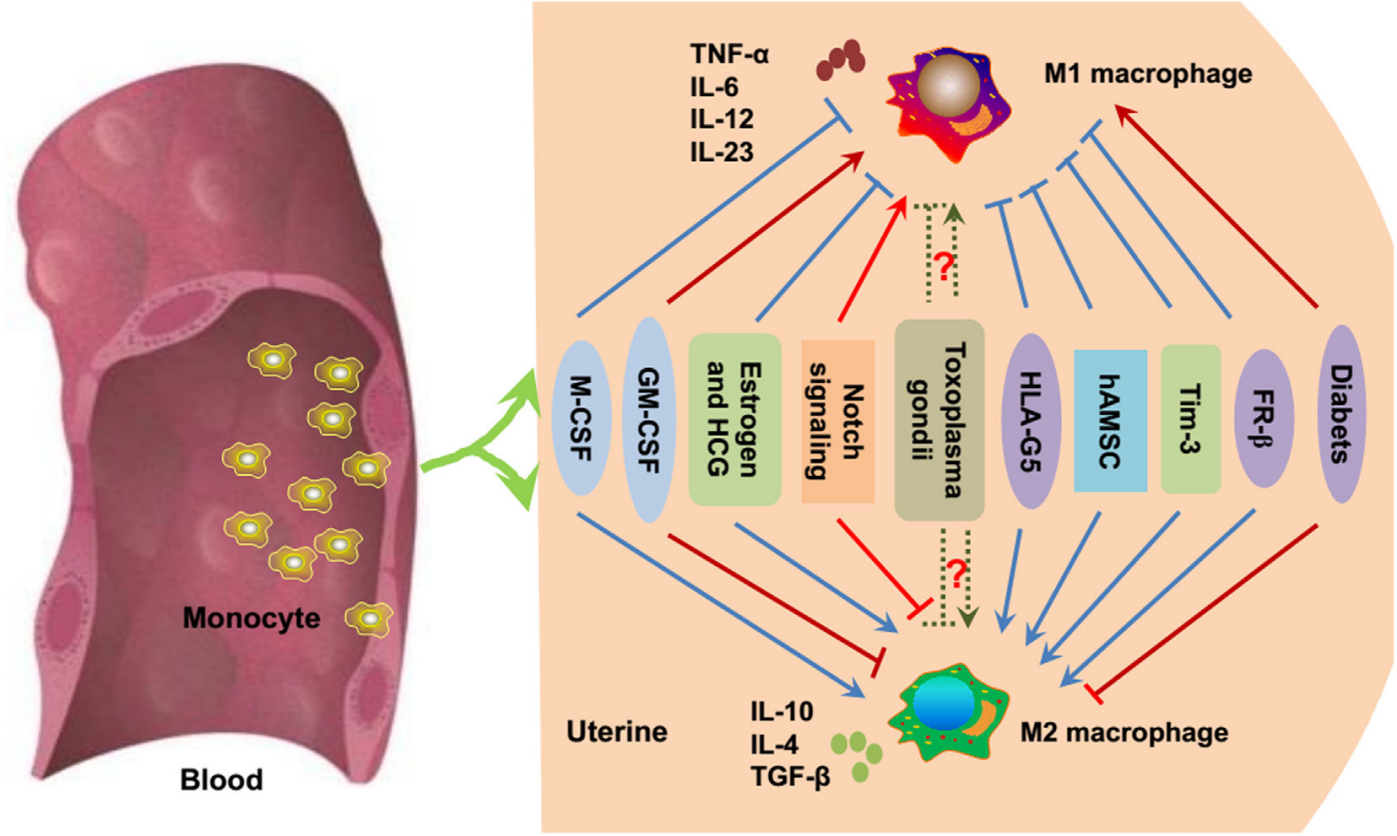
**Essential modulators of macrophage polarization during pregnancy**. Under inflammatory conditions, bone marrow-derived monocytes contribute to tissue macrophage homeostasis. As previously mentioned, M-CSF, estrogen, HCG, HLA-G5, hAMSC, Tim-3, and FR-β promote the polarization toward M2 macrophages (blue point arrows) and inhibit M1 polarization (blue block arrows). GM-CSF, Notch signaling, and diabetes/hyperglycemia have been implicated in the polarization of M1 macrophages (red point arrows), while suppressing M2 macrophage polarization (red block arrows). Whether *Toxoplasma gondii* facilitates M1 or M2 macrophage polarization is uncertain (dashed green arrows), and it mainly depends on the host immune status and the virulence of the pathogen. M-CSF, macrophage colony-stimulating factor; GM-CSF, granulocyte macrophage colony-stimulating factor; HCG, human chorionic gonadotropin; HLA-G, human leukocyte antigen G; hAMSC, human amniotic mesenchymal stem cell; Tim-3, T-cell Ig and mucin domain protein 3; FR-β, folate receptor β.

As mentioned above, the unique macrophage phenotype and heterogeneity are important for the establishment and maintenance of a successful pregnancy. The environment in which macrophages mature and differentiate during pregnancy is important for macrophage polarization. Various secreted cytokines, chemokines, growth factors, and hormones, as well as interactions with related cells, are important for macrophages to acquire their unique phenotype and function (41) (Figure 2).

## GM-CSF and M-CSF

Granulocyte macrophage colony-stimulating factor and M-CSF belong to the CSF family and are major differentiation growth factors that mediate M1 and M2 polarization, respectively. Both GM-CSF and M-CSF as well as their receptors are present at the maternal–fetal interface. The endometrium, decidua, and trophoblast are the main sources of CSFs. Their receptors have been observed in both murine and human female trophoblasts and reproductive tracts (42–46). The membrane receptors have also been identified on endometrial immune cells, such as macrophages, granulocytes, and dendritic cells (42). Endometrial GM-CSF production was shown to be positively regulated by estrogen and inhibited by progesterone (47). With coitus, GM-CSF expression is increased by TGF-β in the male seminal fluid (48). This surge in GM-CSF production together with other cytokines and chemokines induced by seminal fluid triggers a controlled inflammatory response within the decidua associated with an influx of macrophages, which maintains a mild pro-inflammatory phenotype around the time of embryo implantation (49). Svensson et al. (50, 51) found that trophoblast-derived M-CSF polarized maternal monocytes toward M2 macrophages with a resemblance to decidual macrophages during the first trimester of normal pregnancy. GM-CSF-stimulated macrophages presented a phenotype that was more similar to that of macrophages activated by LPS and IFN-γ, namely, M1 activation (50). Among the Th2 cytokines (e.g., IL-4, IL-13), only IL-10 was able to overcome the effect of GM-CSF during macrophage polarization. Therefore, trophoblast-derived M-CSF and IL-10 induce the polarization of decidual macrophages, contributing to the homeostatic and tolerant immune environment required for successful fetal development. Apart from the trophoblast, first trimester decidual cells (FTDCs) are another source of M-CSF and GM-CSF. Li et al. (52) found that FTDC-secreted M-CSF induced decidual immune tolerance by switching to M2 macrophage polarization and phagocytic capacity in response to pro-inflammatory stimuli. However, excessive pro-inflammatory cytokines, such as IL-1β and TNF-α, markedly enhance GM-CSF expression in FTDC, which subsequently polarizes macrophages toward the M1 subtype in PE (53–55). Conversely, enhanced expression of GM-CSF might contribute to PE by promoting M1 polarization.

## Pregnancy-Related Hormones

Apart from immune adaptation, pregnancy is marked by significant temporal changes of a variety of hormones throughout gestation. Therefore, the success of pregnancy might depend on a synchronized immune-endocrine crosstalk at the maternal–fetal interface (56). Hormones are important in terms of maintenance of the suitable environment and sufficient nutrition for the developing fetus. Hormones modulate both innate and adaptive immune cells to adopt to fetal development. Therefore, maternal tolerance to the semiallogeneic fetus is achieved in concert with a variety of endocrine stimulations. Estrogens, progesterone, and human chorionic gonadotropin (HCG) are three of the main hormones during pregnancy. These hormones have recently been proposed to modulate macrophage polarization during pregnancy.

Estrogens are a group of compounds known for their importance in the estrus cycle of humans and other animals. Three main common estrogens are present throughout pregnancy, including estrone (E1), estradiol (E2), and estriol (E3). The placenta is the primary site of E1 and E2 production, and it converts 16-hydroxydehydroepiandrosterone to E3. With estrogen receptors (ERs) expressed in lymphocytes, macrophages, and dendritic cells, estrogens contribute to fetal tolerance by regulating the phenotype and function of different immune cell populations (57). E2 occurs in high concentrations in non-pregnant as well as pregnant females and is responsible for the majority of the “classic” estrogenic effects in reproductive tissues. E2 has bipotential effects on macrophages, with low concentrations promoting pro-inflammatory cytokine production (e.g., IL-1β, IL-6, and TNF-α) and high doses reducing secretion of these cytokines (58). This finding may indicate that E2 can regulate macrophage polarization to some extent. E3 is produced in high concentrations by the fetoplacental unit during pregnancy and accounts for almost 90% of all estrogens produced during pregnancy (59). However, the immunological effects of E3 have not been well characterized, and it is assumed that the effects of E3 are broadly the same as E2 because both estrogens signal through the same ERs (60).

Progesterone is produced by the corpus lutea in the ovaries in non-pregnant females. After conception, progesterone is produced by the *corpus luteum* until 5–6 weeks of gestation, and then after the 12th week of gestation, the placenta becomes the dominant producer of progesterone. Progesterone has various functions, such as promoting endometrial decidualization for embryo implantation (61), inhibiting smooth muscle contractility, and maintaining myometrial quiescence (62). Additionally, progesterone is considered to be anti-inflammatory. Progesterone receptors have been identified in macrophages, and progesterone can inhibit nitrite and NO production as well as TNF-α expression by murine macrophages (63). However, macrophage polarization might not be influenced by progesterone during pregnancy. Furcron et al. (64) found that vaginal progesterone treatment had anti-inflammatory effects at the murine maternal–fetal interface. Inflammation has been implicated in physiological (65) and pathological parturition (66), such as PTL. Therefore, the effects of progesterone in the prevention of PTL may be mediated by its anti-inflammatory capacity (64). Although vaginal progesterone reduces the proportion of decidual macrophages, it does not result in M1 → M2 macrophage polarization in murine models. However, whether progesterone modulates macrophage polarization in human pregnancy is still unclear.

Human chorionic gonadotropin is the first hormone that participates in the interactions between the mother and the fetus. HCG is a heterodimeric glycoprotein, which is initially produced in the developing placenta after conception and later by the placental component syncytiotrophoblast (67). Temporal fluctuations in the production of HCG are marked by its maximal levels by the 10th week of pregnancy, and then it falls slowly to the lowest point at 17 weeks and remains at a low but readily measurable level for the remainder of the pregnancy (68). HCG receptors are widely expressed in reproductive tissues (69), maternal–fetal tissues (70), and immune cells (71, 72). Therefore, HCG is thought to be involved in preserving the progesterone-producing *corpus luteum* (73), promoting angiogenesis (74) and trophoblast differentiation (75) and maintaining myometrial quiescence (76) and maternal–fetal tolerance (77). Macrophages express HCG receptors throughout gestation (78). HCG treatment of IFN-γ-primed macrophages resulted in increased production of NO, reactive oxygen species, and IL-6 and enhanced phagocytosis of apoptotic cells (71). Therefore, HCG can enhance macrophage function (71). At the maternal–fetal interface during late gestation, HCG has anti-inflammatory effects and prevents endotoxin-induced PTL but causes dystocia and fetal compromise in mice (79). Unlike progesterone, HCG treatment reduces the proportion of macrophages at the maternal–fetal interface but induces M1 → M2 macrophage polarization (79).

## Notch Signaling

The Notch signaling pathway is evolutionarily conserved and is involved in regulating cell proliferation, apoptosis, and cell fate decisions during development and adult tissue homeostasis (80). In mammals, there are four Notch receptors (Notch 1–4) and five distinct ligands [Jagged1, Jagged2, Delta-like 1 (DLL1), DLL3, and DLL4]. The interaction between Notch ligands and receptors leads to proteolytic cleavage of the receptor and liberates the Notch intracellular domain (NICD) from the membrane. Then, NICD transfers to the nucleus, where it activates the recombining binding protein suppressor, subsequently allowing the recruitment of coactivators and leading to the transcription of Notch genes. Notch signaling has been proven to determine the fate of immune cells and is involved in T- and B-cell activation (81) and macrophage polarization toward M1 cells (82). Notch signaling is also involved in the polarization of decidual macrophages. Notch signaling pathways exert effects throughout pregnancy and are activated in response to TLR ligands (83). PTL can be induced in animal models by pathogen-derived TLR ligands for TLR4 (LPS), TLR2 (peptidoglycan, PGN), and TLR3 [polyinosinic:cytidylic acid, poly(I:C)] as well as in a synergistic manner (TLR2 + TLR3). Altered expression of Notch signaling-related molecules was closely associated with LPS-induced PTL but not in hormonally induced PTL (83). In the decidua of LPS-induced PTL, macrophage polarization is skewed toward M1 cells, and this process is dependent on the activation of Notch signaling. Furthermore, PGN + poly(I:C) administration induces the expression of DLL-1 and Notch 1 in decidual macrophages, which are double positive for CD11c (M1 marker) and CD206 (M2 marker), with the generation of both M1-associated cytokines (IL-6, TNF-α) and M2-associated cytokines (IL-10). However, lower secretion of both M1- and M2-associated cytokines was observed by the Notch inhibitor gamma-secretase inhibitor (84). Therefore, upregulated Notch-related inflammation may be associated with inflammation-induced PTL by regulating macrophage polarization.

## *Toxoplasma* *gondii*

*Toxoplasma gondii* infection is the leading cause of fetal IUGR among the five pathogens termed TORCH (including *Toxoplasma*, rubella virus, cytomegalovirus, and herpes virus and other pathogens). *T. gondii* infection may result in congenital toxoplasmosis, miscarriage, stillbirth, and increased pregnancy complications (85). All these abnormal pregnancy outcomes may result from immune imbalances induced by *T. gondii* (86). Macrophages are important effector cells for the control and killing of intracellular *T. gondii*, and they also serve as long-term host cells for the replication and survival of the parasite (87). These different outcomes might depend on macrophage activation (M1 or M2) after *T. gondii* infection. Jensen et al. (88) found that macrophages infected with the type I and type III *T. gondii* polarized to M1 activation, while type II infection skewed to M2 polarization in pregnant mice. Moreover, the ability of *Toxoplasma* to induce specific macrophage activation could be associated with consequences on virulence, local parasite burden, and inflammatory-related pathology. For example, macrophages infected with the TgCtwh6 strain (type I with low virulence) were preferentially biased toward M1 activation and an increased trophoblast apoptosis index *in vitro* (22), while TgCtwh3 (type I with high virulence)-infected macrophages were polarized toward M2 activation. However, higher apoptosis levels of trophoblasts were found in TgCtwh3 infection *in vivo*, which might have resulted from further Th2 bias by TgCtwh3, subsequently promoting parasite duplication (22).

Parasite-derived factors, ROP16 and GRA15, work independently to achieve M1 and M2 activation (22). ROP16, a rhoptry protein, has serine–threonine kinase activity (89) and induces M2 activation through the STAT6 pathway, while GRA15 drives macrophages to M1 polarization *via* NF-κB activation. Further investigations showed that the induction of macrophage polarization depends on polymorphisms of the two proteins in strains with different genotypes (90, 91). The phosphokinase ROP16_I/II_ allelic variation of leucine but not serine at 503 is responsible for M2 polarization, while GRA15_II_ promotes M1 polarization (92). Differing from the archetypal lineages of type I, II, and III circulating in Northern America and Europe, type Chinese 1 is the predominant clonal lineage in China. The Wh6 strain of Chinese 1 has comparatively low virulence to mice (93). Sequencing of the effectors of ROP16 and GRA15 showed that Wh6 strain possesses the allelic polymorphisms of the two effector molecules (ROP16_I/III_ 503L and GRA15_II_), suggesting a different mechanism of macrophage-biased induction in Chinese 1 strain infection (85). Rats with acute Wh6 infection prior to pregnancy-favored M1 polarization, accompanied by an increased proportion of fetal IUGR, inflammatory scores of the placenta, and reduced numbers of embryos (85). These findings strongly suggest the association of M1-biased immunity induced by *Toxoplasma* infection on gestation with the consequence of immunopathology and adverse pregnancy outcomes. However, an M2 bias was observed in acute infection after gestation, indicating that part or most of the macrophages might be induced to M2 in the microenvironment during pregnancy, and the Th2-dominant immune response in pregnant rats somewhat inhibits the excessive bias of the macrophages toward M1. Most of these findings were observed in pregnant rats infected with *T. gondii*, and the macrophages were obtained from the peritoneal cavity. The relationship between *T. gondii* infection and macrophage polarization during human pregnancy is still unclear.

## Human Leukocyte Antigen G

Human leukocyte antigen G belongs to HLA class Ib. There are seven isoforms of HLA-G; G1–4 are membrane bound, whereas G5–7 are soluble proteins (94). Both membrane-bound and soluble HLA-G molecules are detected in human placentas, decidua, and maternal blood (95). During healthy pregnancy, the plasma level of soluble HLA-G5 increases in the first trimester and gradually declines as the pregnancy advances (96). A decreased or undetectable level of soluble HLA-G in the maternal circulation during the first/second trimester is associated with complications, such as recurrent spontaneous abortion, PE, and IUGR (97, 98). Soluble HLA-G5 participates in immune tolerance under physiological (pregnancy) and pathological (tumor and allograft) conditions. Therefore, decidual macrophage maturation and differentiation might be regulated by HLA-G5, which could be released from the trophoblast, as its functional receptors are expressed in monocytes and decidual macrophages (94, 98). Lee et al. (99) demonstrated that soluble HLA-G5 polarized macrophages toward the M2 phenotype, with higher phagocytic activity and increased IDO expression, suppressing IFN-γ expression in T-cells and promoting trophoblast invasion. Therefore, these findings suggest a role for soluble HLA-G5 in driving macrophage polarization into the decidual macrophage-like phenotype, which promotes maternal–fetal tolerance and placental development. The potential molecular mechanisms that regulate the soluble HLA-G5-polarized macrophages in maternal–fetal tolerance and placental remodeling are still unclear.

## Amniotic Mesenchymal Stromal Cell

Mesenchymal stem/stromal cells (MSCs), derived from both maternal and fetal compartments, strongly contribute to maternal–fetal tolerance, mainly resulting from their broad immune regulatory capacities (100–102). The immune regulatory properties of human amniotic MSCs (hAMSCs) are the subject of growing interest (103, 104). In addition to T lymphocytes, hAMSCs also act on the monocyte/macrophage lineage regulating their activation (105–107). Interestingly, hAMSCs promote monocyte differentiation into anti-inflammatory M2 cells (107). Indeed, hAMSCs from a normal pregnancy block M1 differentiation and switch them to M2 cells (108). Therefore, it is reasonable to speculate that the immune-modulatory properties of hAMSCs are altered and contribute to the development of abnormal pregnancies, such as PE. However, no intrinsic impairment of hAMSCs was found between healthy pregnancy and PE (108). These results suggest that hAMSCs might not contribute to the development of PE but conversely, could participate in offsetting the inflammatory status that characterizes PE.

## T-Cell Immunoglobulin and Mucin Domain Protein 3 (Tim-3)

T-cell immunoglobulin and mucin domain protein 3 was first described as a molecule specifically expressed on the surface of IFN-γ-producing Th1 and cytotoxic T-1 cells (109). The engagement of Tim-3 with its ligand, galectin-9, could induce the exhaustion or apoptosis of effector T cells, and thus might regulate immune tolerance (110). In addition to being expressed on activated T-cells, Tim-3 is constitutively expressed on cells of the innate immune system in both mice and humans, and Tim-3 expression is enhanced in M2 macrophages (111, 112). Recent data have demonstrated that Tim-3 regulates innate immune cells to induce maternal–fetal tolerance (113, 114). Chabtini et al. (114) found that blockade of Tim-3 by RMT3-23 (anti-Tim-3) antibody resulted in accumulation of macrophages at the maternal–fetal interface and upregulation of pro-inflammatory cytokines. Furthermore, Tim-3 blockade during early pregnancy inhibits the phagocytic potential of macrophages, resulting in the accumulation of apoptotic bodies at the maternal–fetal interface. This accumulation elicits local immune responses, leading to the abrogation of tolerance at the maternal–fetal interface and fetal rejection. All these findings suggest that Tim-3 blockade during the first trimester skews macrophages toward M1 activation rather than M2 polarization.

## Folate Receptor β

Hofbauer cells are macrophages that reside within the mesenchymal stroma of the chorionic villi (115), which are thought to be of fetal origin (116). Constitutive expression of CD209 and high levels of CD163, CD45, HLA-A, HLA-B, HLA-C, IL-10, and TGF-β suggest that Hofbauer cells skew toward M2 in healthy pregnancies (117, 118). Therefore, Hofbauer cells may participate in placental angiogenesis, tissue remodeling, and modulation of inflammation-like decidual macrophages (118). Folate receptors (FRs) are glycoproteins responsible for high affinity folate binding and subsequent transport into cells *via* endocytosis. The FR family includes three types: FR-α, FR-β, and FR-γ/γ′. The expression profile of each FR subtype depends on differentiation stage and tissue type. FR-β is expressed on a number of hematopoietic precursor cells and myelomonocytic lineages. However, it usually stays in an inactive form, unable to bind folate. Functional FR-β is detected on activated macrophages in the placenta. Moreover, it is preferentially expressed on M2 macrophages and is considered a biomarker for M2 macrophages (119). Decreased expression of FR-β and CD163 has been observed in Hofbauer cells from women with PE (120). These findings indicate that Hofbauer cells might switch toward M1 polarity in PE, then M1 macrophages might contribute to the development of PE.

## Diabetes/Hyperglycemia

Diabetes impairs fetal development and increases the risk of metabolic disorders in adulthood. Strong changes in the expression of placental genes related to markers and mediators of inflammation are elicited by diabetes (121). Compared with healthy pregnant women, Hofbauer cells exhibit an M1-like phenotype and function in women with diabetes (122). Further investigation proved that diabetes and/or hyperglycemia could switch Hofbauer cells from the M2 to M1 phenotype *in vivo* and *in vitro*. Therefore, the altered functional phenotype of Hofbauer cells might contribute to the detrimental inflammation status of the placenta and eventually result in negative consequences to fetal development. Although the underlying mechanism is still unclear, there might be an assumption that the shift from the M2 to the M1 profile might involve another higher level of communication between environmental stimuli and cell responses, such as epigenetic modifications.

In addition, other factors modulating macrophage polarization have been found in other immune disorders (123–126), such as programmed cell death 1, IRGM, and miRNAs. However, whether these modulators contribute to macrophage homeostasis during pregnancy has not been characterized.

## The Bright Future of Macrophage-Originated Therapy for Pregnancy Complications

It is now known that macrophage polarization governs the fate of an organ (127). As discussed above, accurately regulated uterine macrophage polarization, namely, the M1/M2 balance, is involved in the establishment, maintenance, and termination of normal pregnancy. However, an M1/M2 imbalance results in pregnancy loss or pregnancy complications. Thus, the realization that macrophages play a pivotal role in directing pregnancy outcomes, either directly or by influencing T- and B-cell functions, is opening novel approaches to pregnancy complications using immunotherapy.

Modulating macrophage polarization, namely, converting the M1-type macrophages into M2-type macrophages, might be a breakthrough that will facilitate successful immunotherapy. Although no studies have focused on pregnancy, the combination of 5-Aza 2-deoxycytidine and Trichostatin A, two epigenetic modifiers, decreased expression of the M1 phenotype while augmenting expression of the M2 phenotype in LPS-induced macrophages (128). It has also been found that a shift toward M2-like macrophages protects against structural and functional damage in adriamycin-induced nephropathy in SCID mice (129).

Although the abovementioned modulators of macrophage polarization have only recently received attention from researchers, they do have a promising future. Using the abovementioned studies as examples, more studies utilizing modulators to manipulate the pathogenesis of disorders should be undertaken. Given that those modulators contribute to the development of inflammatory disorders, they could also serve as more effective therapeutic approaches (127). Furthermore, we believe that macrophage-based immunotherapy will help ameliorate inflammatory disorders *via* more natural, effective, and less-toxic and disabling means than drugs or surgery.

## Conclusion

Macrophages play important roles in embryo implantation, placentation, pregnancy maintenance, and initiation of parturition. With high plasticity, their phenotypes and functions are influenced by the microenvironment. Accurate regulation of macrophage polarization is required for successful pregnancy. Otherwise, pregnancy complications and poor outcomes occur with ill-timed or ill-placed macrophage polarization. Although several factors regulating M1 versus M2 polarization during pregnancy have been found, such as growth factors, hormones, infection, and Tim-3, numerous questions remain: (i) What are the specific M1 and M2 factors and their roles in human pregnancy? (ii) Since M2 macrophages facilitate pregnancy maintenance, how is this accomplished at the molecular and cellular levels? (iii) How do polarized macrophages influence pregnancy at the cellular and molecular levels? (iv) As pregnancy is characterized by multiple stages, are there any specific factors modulating macrophage polarization at specific stages? and most importantly, (v) Can small molecules be developed to switch or regulate macrophage polarization?

## Author Contributions

Y-HZ: design, text and drawings. MH and YW: text. A-HL: design, text revision and final approval.

## Conflict of Interest Statement

The authors declare that the research was conducted in the absence of any commercial or financial relationships that could be construed as a potential conflict of interest.

## Abbreviations

APC, antigen-presenting cell; DLL, delta-like; E1, estrone; E, estradiol; E3, estriol; ER, estrogen receptor; EVT, extravillous trophoblast; FR, folate receptor; FTDC, first trimester decidual cell; Gal-9, galectin-9; GM-CSF, granulocyte macrophage colony-stimulating factor; GSI, gamma-secretase inhibitor; hAMSC, human amniotic MSC; HCG, human chorionic gonadotropin; HLA-G, human leukocyte antigen G; iNOS, inducible nitric oxide synthase; IFN-γ, interferon-gamma; IL, interleukin; IKK, inhibitor of NF-κB kinase; IUGR, intrauterine growth restriction; JAK, janus kinases; JNK, c-Jun N-terminal kinase; LPS, lipopolysaccharides; MAPK, mitogen-activated protein kinase; MHC, major histocompatibility complex; M-CSF, macrophage colony-stimulating factor; MSC, mesenchymal stem/stromal cell; NF-κB, nuclear factor κB; NICD, Notch intracellular domain; NO, nitric oxide; poly(I:C), polyinosinic:cytidylic acid; PE, preelampsia; PGN, peptidoglycan; PTL, preterm labor; ROS, reactive oxygen species; STAT, signal transducers and activators of transcription; TAM, tumor-associated macrophage; *T. gondii, Toxoplasma gondii;* Th, T helper; Tim-3, T-cell immunoglobulin and mucin domain protein 3; TLR, toll-like receptor; TNF-α, tumor necrosis factor alpha; TGF-β, transforming growth factor beta.
